# Magnolol as a potent antifungal agent inhibits *Candida albicans* virulence factors *via* the PKC and Cek1 MAPK signaling pathways

**DOI:** 10.3389/fcimb.2022.935322

**Published:** 2022-07-22

**Authors:** Yufei Xie, Hong Hua, Peiru Zhou

**Affiliations:** Department of Oral Medicine, Peking University School and Hospital of Stomatology, National Center of Stomatology, National Clinical Research Center for Oral Diseases, National Engineering Research Center of Oral Biomaterials and Digital Medical Devices, Beijing, China

**Keywords:** magnolol, *Candida albicans*, virulence factor, antifungal mechanism, PKC, Cek1, MAPK signaling pathway

## Abstract

Magnolol, a lignin compound extracted from *Magnolia officinalis* Cortex, has been found to have prominent antifungal effects against *Candida albicans*. However, the specific mechanism still remains unclear. Therefore, this study aimed to further explore the inhibition mechanism of magnolol against *Candida albicans* virulence factors and the related signaling pathways. By an XTT reduction assay, a hyphal formation assay, confocal laser scanning microscopy, transmission electron microscopy, a calcofluor white staining assay, and a cell wall β-glucan quantitative detection assay, we evaluated the inhibitory effects of magnolol against the adhesion, hyphal formation, biofilm viability, biofilm spatial structure, and cell wall ultrastructure of *Candida albicans*. Moreover, by RNA sequencing and qRT-PCR, we confirmed the effects of magnolol in inhibiting the gene expression of *Candida albicans* virulence factors and the related signaling pathways. The results revealed that the adhesion and hyphal formation of *Candida albicans* were inhibited significantly by magnolol. The viability and spatial structures of *Candida albicans* biofilms were further weakened. *Candida albicans* ultrastructure showed partial thinning of cell walls and even rupture, with cytoplasmic leakage. The cell wall intergrity and β-glucan content were also radically reduced. Moreover, magnolol caused significant inhibition of the expression of *Candida albicans* adhesion, invasion, hyphal formation, biofilm formation, β-1,3-glucan synthesis, and hydrolase secretion-related genes, including *ALS1*, *ALS3*, *EFG1*, *EAP1*, *FKS1*, *FKS2*, *PLB2*, and *SAP2*. Furthermore, the PKC pathway-related genes (*RHO1*, *PKC1*, *BCK1*, *MKK2*, *MKC1*) and Cek1 pathway-related genes (*CDC42*, *CST20*, *STE11, HST7*, *CEK1*) were also significantly downregulated, indicating that the inhibition of magnolol against *Candida albicans* virulence factors might be related to PKC and Cek1 MAPK signaling pathways. In conclusion, the findings of this study confirmed the inhibition mechanism of magnolol against *Candida albicans* virulence factors, which might be related to PKC and Cek1 MAPK pathways, thus laying the theoretical foundation for its clinical antifungal applications.

## Introduction

Oral candidiasis, one of the most common opportunistic infections, caused mainly by *Candida albicans*, has seen a significant increase in prevalence due in part to the widespread application of broad-spectrum antibiotics, immunosuppressants, and glucocorticoids. Long-term oral candidiasis can lead to severe deep fungal infections, even inducing cancer and endangering life (Cannon, 2022). The mortality rate of invasive candidiasis can reach 40–60% (McCarty et al., 2021). The widespread use of traditional antifungal drugs, including azoles, echinocandins, and polyenes, has led to increasingly severe antifungal resistance (Berman and Krysan, 2020). In addition, traditional antifungal agents may induce severe adverse reactions, such as hepatotoxicity, nephrotoxicity, and gastrointestinal reactions (Houšť et al., 2020; Xiao et al., 2022). The total incidence of adverse reactions of topical antifungal agents for oral candidiasis in immunocompetent patients with fluconazole can even reach 39.5% (Xiao et al., 2022). Therefore, *Candida* infections have become one of the crucial hidden dangers to human health, and the development of safer and more effective antifungal agents is urgently required to address this problem.

*Magnolia officinalis* Cortex (*M. officinalis*), as a primary component of Traditional Chinese Medicine formulae, was widely used for the treatment of infectious diseases associated with pathogenic microorganisms, such as constipation, diarrhea, asthma, phlegm, and malaria (Lin et al., 2021; Niu et al., 2021). The major constituents in the extract of *M. officinalis* Cortex are magnolol and honokiol, which are isomers with hydroxylated biphenol compounds (Phan et al., 2022). Magnolol, a lignin compound, can exert multiple pharmacological effects such as anti-inflammatory, antioxidant, antibacterial, and antifungal activities (Zhang et al., 2019). Studies have shown that magnolol has significant antibacterial effects on *Streptococcus mutans*, *Porphyromonas gingivalis*, and *Fusobacterium nucleatum*, which are salivary bacteria responsible for dental caries, periodontosis, and oral malodor (Greenberg et al., 2007; Chiu et al., 2021). As for the antifungal activities, early studies showed that magnolol could inhibit *C. albicans* azole efflux and produce synergistic effects with fluconazole by competing with ATP binding cassette transporter Cdr1p substrates (Sun et al., 2015b). However, researches on the antifungal mechanism of magnolol are still limited.

To further evaluate magnolol’s antifungal effects in oral candidiasis, we measured its MIC (the minimum inhibitory concentration against planktonic *Candida* strains) and BMIC_90_ (the minimum inhibitory concentration inhibiting 90% *Candida* biofilms) against multiple *Candida* spp. in our previous study, and fluconazole was used as a control standard substance (Zhou et al., 2017). The MIC and BMIC_90_ of magnolol for *C. albicans* were determined as 40 and 160 μg/mL, respectively. When the concentration exceeded MIC and BMIC_90_, magnolol could exert fungicidal effects on *C. albicans* planktonic and biofilm conditions, repectively, whereas at lower concentrations, magnolol could mainly impact the virulence phenotypes of *C. albicans*. And since oral candidiasis is mainly caused by *C. albicans* biofilms, we chose the BMIC_90_ of magnolol in the present study to further explore its anti-*Candida* mechanism.

The pathogenicity of *C. albicans* mainly depends on its virulence factors, such as adherence and invasion, hyphal and biofilm formation, cell wall integrity, and hydrolase secretion (Staniszewska, 2020). The cyclic AMP (cAMP) and mitogen-activated protein kinase (MAPK) signaling pathways has further been found to play significant roles in regulating the expression of various virulence factors (de Dios et al., 2010; Huang et al., 2019). The cAMP signaling pathway is crucial in regulating *C. albicans* morphogenesis and environmental sensing (Huang et al., 2019). The MAPK signaling pathways, including PKC, Cek1, and HOG pathways, are also significant in the regulation of *C. albicans* hyphal formation, biofim formation, and the adaptation to cell wall stress (Schoeters and Van Dijck, 2019). Previous studies have shown that a variety of natural bioactive compounds, such as sanguinarine, linalool, and berberine, can inhibit *C. albicans* virulence factors *via* cAMP and MAPK pathways (Hsu et al., 2013; Zhong et al., 2017; Huang et al., 2021). However, whether magnolol can affect *C. albicans* virulence factors and the associated signaling pathways is still unclear. Therefore, this study focused on the above issues to further clarify the anti-*Candida* mechanism of magnolol.

## Materials and methods

### Drug and medium preparation

Standard substances of magnolol (Batch number: 110729-202015) were obtained from the National Institutes for Food and Drug Control (Beijing, China). The purity of magnolol was measured using high-performance liquid chromatography and determined to be about 98.8%. The drugs were dissolved in dimethyl sulfoxide (Sigma-Aldrich Co., St Louis, MO, USA) and stored at a concentration of 1.28 × 10^5^ μg/mL at –80°C, following the broth dilution testing reference method M27-A3, as recommended by the Clinical and Laboratory Standards Institute (CLSI) (Wayne and Clinical and Laboratory Standards Institute, 2008).

Roswell Park Memorial Institute (RPMI) 1640 medium containing L-glutamine (Life Technologies Co., Madison, WI, USA) was buffered to pH 7.0 using 0.165 M 3-morpholinopropane-1-sulfonic acid (Sigma-Aldrich Co.). Yeast nitrogen base medium (YNB)-50 G and YNB-100 G were prepared using YNB medium (Beijing Solarbio Science and Technology Co. Ltd., Beijing, China) supplemented with 50 and 100 mM glucose, respectively.

### *Candida* strains and culture conditions

The standard *C. albicans* strain (ATCC 90028) was purchased from the American Type Culture Collection (ATCC; Manassas, VA, USA). The strain was stored in 20% glycerol and frozen at –80°C. Before the experiment, the strains were cultured on Sabouraud dextrose agar (SDA) plates (BioMérieux Industry Co. Ltd., Marcy l’ Etoile, France) at 37°C, 48 h and stored at 4°C.

To mimic the process of *C. albicans* adhesion and biofilm formation *in vitro*, yeast cells were subcultured on an SDA plate at 37°C for 18 h, followed by overnight incubation in YNB-50 G medium at 200 rpm. The yeast cells were then harvested and adjusted to 1 × 10^7^ cells/mL in YNB-100 G medium. Following this, the vortexed yeast suspension was added into the wells of microtiter plates or culture dishes, and incubated for 2 h at 37°C. Next, the yeast suspension was aspirated, and magnolol solution was added to achieve final concentrations of 40, 80, 160, and 320 μg/mL, based on the MIC and BMIC_90_ of magnolol obtained from our previous research (Zhou et al., 2017). The wells without drug supplementation were considered the drug-free controls.

### *C. albicans* adhesion assay

*C. albicans* was cultured referring to the method described above and incubated with 160 and 320 μg/mL of magnolol for 4, 6, and 12 h. To quantify the adherence of *C. albicans*, the reduction in XTT was measured according to a previously described protocol (Pierce et al., 2008). Two milliliters of 1 mg/mL XTT sodium salt (Sigma-Aldrich Co.) solution, 100 μL of 0.04 mol/L menadione (Sigma-Aldrich Co.) solution, and 7.9 mL of sterilized phosphate-buffered saline (PBS) were mixed to obtain 10 mL of XTT–menadione–PBS reagent. Aliquots (200 μL) of this reagent were added to each well and incubated at 37°C for 3 h in the dark. Following this, 100 μL of the suspension in each well was transferred to a new 96-well microtiter plate. The optical density (OD) value at 490 nm was measured using a microplate reader (BioTek Instruments, Inc., Winooski, VT, USA). Each experiment was repeated three times, and the mean OD value was calculated to represent the adherence of *C. albicans* cells.

### Hyphal formation assay

The hyphal formation assay of *C. albicans* was performed in RPMI 1640 medium supplemented with 10% fetal bovine serum (FBS). 200 μL of *C. albicans* suspension (1 × 10^6^ cells/mL) was added into 96-well plates and incubated with 10, 20, 40, 80, 160, 320, 640, and 1280 μg/mL of magnolol for 4, 16, and 24 h at 37°C overnight. The quantification of the inhibitory effects of magnolol on hyphal formation was determined by the proportion of individual hyphae and the number of yeast cells under an optical microscope, which was calculated using Image J software (NIH, Bethesda, MD, USA). Each experiment was repeated three times, and the hyphae formation rate (%) was calculated as the number of hyphae/the number of hyphae and yeast cells × 100%.

### Confocal laser scanning microscopy assay

Before the staining step of the confocal laser scanning microscopy (CLSM) assay, *C. albicans* biofilm formation was performed in glass-bottom cell culture dishes (NEST Biotechnology Co., Ltd., Wuxi, China) using the method described above. The biofilms incubated without magnolol were regarded as the live controls, and those incubated with isopropyl alcohol for 60 min were regarded as the dead controls. The magnolol solution was diluted to achieve 80, 160, and 320 μg/mL.

Rapid immunofluorescence staining was performed using the LIVE/DEAD FungaLight Yeast Viability Kit (Molecular Probes, Inc., Eugene, OR, USA). Live yeasts with intact cell membranes were stained fluorescent green by SYTO9, whereas dead yeasts with damaged membranes were stained fluorescent red, indicating the penetration of propidium iodide. According to the kit manufacturer’s protocol, 500 μL of stain solution was added to each biofilm dish and incubated in the dark at room temperature for 30 min. The biofilms were observed under a confocal laser scanning microscope (Carl Zeiss, Inc., Oberkochen, Germany). Then, three-dimensional (3-D) reconstruction was carried out using Leica Application Suite X (LAS X) software. At least three random visual fields were chosen in each dish, and three duplicate culture dishes for each group were observed.

### Transmission electron microscopy assay

For transmission electron microscopy (TEM) assay, *C. albicans* biofilms were first cultured referring to the method described above, and incubated with 160 and 320 μg/mL of magnolol for 24 h. The biofilm cells were then harvested and centrifuged at 12000 rpm for 20 min. After aspirating the supernatant, the sediment at the bottom was fixed in 2.5% glutaraldehyde and postfixed in 1% osmium tetroxide. The pellet was washed by sodium cacodylate buffer, dehydrated with gradient alcohol, replaced by propylene oxide, and embedded in Epon 812. Semithin sections (1 μm) were cut, stained with methylene blue, and then observed by a microscope. Ultrathin sections were stained with uranyl acetate and lead citrate, and then examined under a JEM-1400 transmission electron microscope (JEOL, Ltd., Tokyo, Japan). A specific image system for TEM (CCD camera) was used to analyze the images. At least three random visual fields were chosen in each semithin section, and three duplicate semithin sections for each group were observed.

### Calcofluor white staining assay

The magnolol-induced cell wall stress and inhibition of *C. albicans* were further detected using potassium hydroxide (KOH) and calcofluor white (CFW) staining. *C. albicans* suspension (1 × 10^8^ cells/mL) was cultured referring to the method described above, and incubated with 80, 160 and 320 μg/mL of magnolol at 37°C for 24 h. The samples were mounted on glass slides, stained with 10% KOH and 0.1% CFW, and visualized by fluorescence microscope (Olympus BX51; Olympus, Tokyo, Japan). CFW was excited at 355 nm and detected at 300–440 nm, and the images were taken under the same exposure time. After the normalization by cell number, the relative mean fluorescence intensities were quantified by Image J software (NIH, Bethesda, MD, USA). At least three random visual fields were chosen in each glass slides, and three duplicate for each group were observed.

### Cell wall β-glucan quantitative detection assay

*C. albicans* cell wall β-glucan content was determined using Yeast β-glucan Content Chemical Colorimetric Quantitative Detection Kit (Chen Gong Biotechnology Co., Shanghai, China), following the manufacturer’s instructions and referencing the methods of previous studies with modifications (Tondolo et al., 2017). Briefly, *C. albicans* was cultured referring to the method described above, and incubated with 80, 160 and 320 μg/mL of magnolol at 37°C for 24 h. The cell wall β-glucan of 5 mL *C. albicans* suspension (1 × 10^8^ cells/mL) was extracted using the extraction liquid of the kit and the sediment was diluted by 5 mL of the assay buffer. Each 500 µL of the samples was added by 500 µL of the benzidinediazo-bis-1-naphtylamine-4 sulfonic acid reaction solution, and the absorbance was measured at 540 nm on a microplate reader (BioTek Instruments, Inc., Winooski, VT, USA). The absorbance values were detected and fitted to the standard curve to calculate β-glucan concentrations. Each experiment was repeated three times, and the mean OD value was calculated to represent the cell wall β-glucan content of the *C. albicans* suspension.

### Total RNA extraction, RNA sequencing, and qRT-PCR

Before RNA extraction, *C. albicans* biofilms were cultured referring to the method described above and incubated with 80, 160, and 320 μg/mL of magnolol for 24 h. The biofilm cells were then harvested by centrifugation, and the sediment was flash-frozen in liquid nitrogen. Total RNA was extracted using the TRNzol Universal reagent (Tiangen Biotech Co., Beijing, China) and resuspended in diethyl pyrocarbonate water (Sigma-Aldrich Co.) according to the manufacturer’s protocol. The concentration, purity, and quality of the isolated RNA samples were determined using a Nano-Drop-One Spectrophotometer (Thermo Scientific, Waltham, MA, USA). Construction of the RNA-seq library and sequencing was completed by the BGI company (Wuhan, China). Single-end 1 × 50 bp sequencing was performed on a BGISEQ-500 system. Raw data were processed using CASAVA V1.6 package (Illumina, San Diego, CA, USA). The quality control of each sample was accomplished using FASTQC V0.11.5. Clean reads were aligned to the *C. albicans* reference genome. RSEM was used to calculate gene expression, and differential expression was determined using BGI’s developed algorithm (Li and Dewey, 2011). The statistical significance of differential expression was determined using multiple testing combined with the false discovery rate (FDR). The differential expressed genes (DEGs) with log2 (fold change)  >  1 and FDR  <  0.001 were considered significant. Gene Ontology (GO) analysis was performed using the GO Term Finder to describe the biology of a gene product, including the biological process, cellular component, and molecular function.

For qRT-PCR, the extracted total RNA was reverse transcribed using a cDNA synthesis kit for qRT-PCR (Takara Biotechnology, Dalian, China). The quantitative real-time step was performed using SYBR green PCR kits (Takara Biotechnology) and a 7500 RealTime PCR instrument (Applied Biosystems, Foster City, CA, USA) with reference to the manufacturer’s protocol. Target genes of *ALS1*, *ALS3*, *EFG1*, *EAP1*, *FKS1*, *FKS2*, *PLB2*, *SAP2*, *RHO1*, *PKC1*, *BCK1*, *MKK2*, *MKC1, CDC42*, *CST20*, *STE11*, *HST7*, and *CEK1.* were amplified accordingly. The relative expression levels of the genes were normalized to the expression of 18S rRNA. The fold change was calculated as 2^−ΔΔCt^ (Livak and Schmittgen, 2001). The primer sequences for qRT-PCR are presented in **Table S1**. Each experiment was repeated three times.

### Statistical analysis

The data were analyzed using parametric statistical tests. One-way analysis of variance (ANOVA) was used to analyze the results of the adhesion assay, CFW staining assay, β-glucan quantitative detection assay, and qRT-PCR. For the correlation analysis of the hyphal formation assay, Pearson correlation was applied. The independent-samples *t*-test was used to analyze the relative fold-change in gene expression between the magnolol-treated and control groups. Statistical Product and Service Solutions (SPSS) 23.0 software (IBM Corp., Armonk, NY, USA) was used for statistical analysis. *P* < 0.05 was considered statistically significant.

## Results

### Magnolol decreased the adherence of *C. albicans*

**Figure 1:**
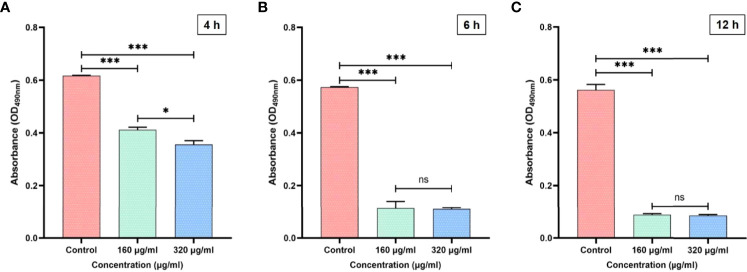
**(A)** The inhibitory effects of magnolol against *C. albicans* adhesion at 4 h. **(B)** The inhibitory effects of magnolol against *C. albicans* adhesion at 6 h. **(C)** The inhibitory effects of magnolol against *C. albicans* adhesion at 12 h. *, *P* < 0.05; ***, *P* < 0.001; ns, no significance.

Adhesion is the primary virulence factor of *C. albicans* and the crucial first step of *Candida* biofilm formation (Livak and Schmittgen, 2001). To evaluate the adherence of *C. albicans*, an XTT reduction assay was performed, and three time-points (4, 6, 12 h) were chosen based on the initial stages of biofilm formation (0–12 h) (Lohse et al., 2018). At the three time-points, the *C. albicans* adherence was significantly inhibited by magnolol at a drug concentration of 160 or 320 μg/mL (**Figure 1**). When magnolol treatment time was prolonged from 6 to 12 h, the number of adherent *C. albicans* decreased significantly at a drug concentration of 160 μg/mL, and was maintained at a similar low level when the drug concentration reached 320 μg/mL. At the 12 h time-point, magnolol could inhibit 84.3% of *C. albicans* from adhering at a drug concentration of 160 μg/mL, and 84.7% at a drug concentration of 320 μg/mL.

### Magnolol inhibited the hyphal formation of *C. albicans*

**Figure 2:**
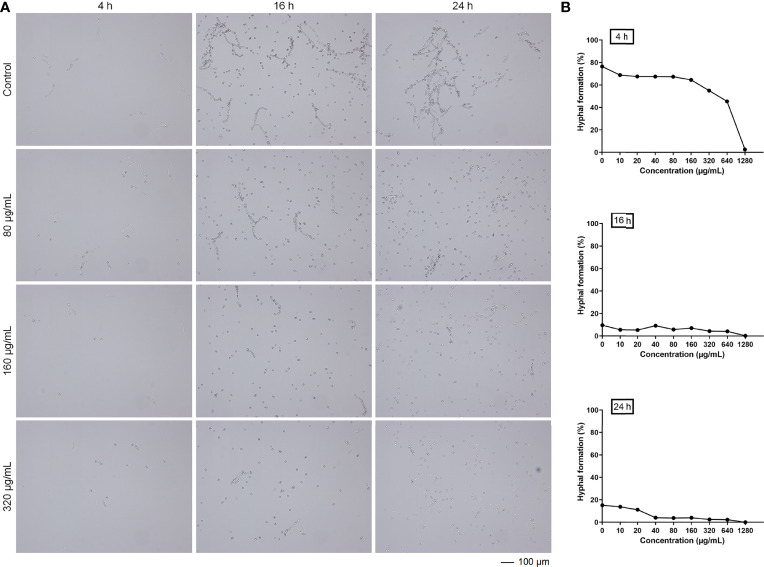
**(A)** The inhibitory effects of magnolol against *C. albicans* hyphal formation at different time-points. **(B)** The trend in *C. albicans* hyphal formation rates exposed to various magnolol concentrations.

In the process of *Candida* biofilm formation, the stage following adhesion is hyphal formation (12–24 h), which is another key virulence factor of *C. albicans*, leading to host invasion and disseminated infection (Wall et al., 2019). Therefore, the inhibitory effects of magnolol against the hyphal formation were analyzed under the microscope and further calculated using Image J software. After 4 hours of drug application, magnolol significantly inhibited cell proliferation and hyphal formation of *C. albicans*, and with the increase of magnolol concentration, its inhibitory effect gradually enhanced. (**Figure 2A**). With the extension of the drug action time, the proportion of hyphae decreased remarkably from 16 h to 24 h. The results above reveal that magnolol could significantly inhibit the hyphal formation of *C. albicans*, with a negative correlation at the 16 h time-point between the drug concentrations and the hyphal formation rate (r = –0.838) (**Figure 2B**).

### Magnolol weakened the viability and spatial structure of *C. albicans* biofilm

**Figure 3:**
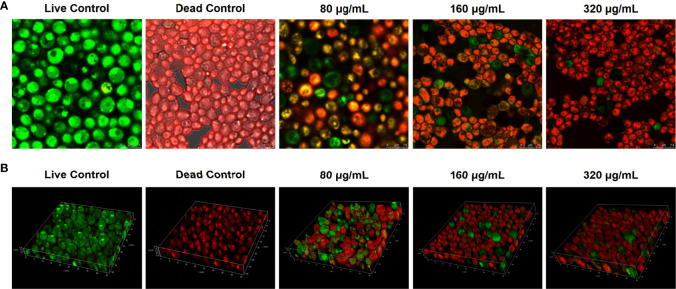
**(A)** The evaluation of the inhibitory effects of magnolol against *C. albicans* biofilm viability using CLSM. **(B)** The biofilm composition and spatial structure of *C. albicans* under the effect of magnolol by 3-D reconstruction.

*C. albicans* can enhance its resistance to antifungal agents and host immunity through the critical virulence factor of biofilm formation (24 h); thus, the inhibitory effects of magnolol against *C. albicans* biofilms were further verified using CLSM (Cavalheiro and Teixeira, 2018). The gross number of dead *C. albicans* cells in the magnolol-treated groups increased significantly compared with that in the live control groups. The results suggest that magnolol treatment could significantly inhibit the viability of *C. albicans* biofilms (**Figure 3A**). To observe the weakening effect of magnolol against the spatial structures of *C. albicans* biofilms, 3-D reconstruction was further performed. Compared with the live control groups, the *C. albicans* biofilms of the magnolol-treated groups displayed a poorly developed architecture and loosely packed cells (**Figure 3B**). These results indicate that magnolol could inhibit *C. albicans* biofilm formation and damage the spatial structures.

### Magnolol reduced *C. albicans* cell wall integrity and β-Glucan content

**Figure 4:**
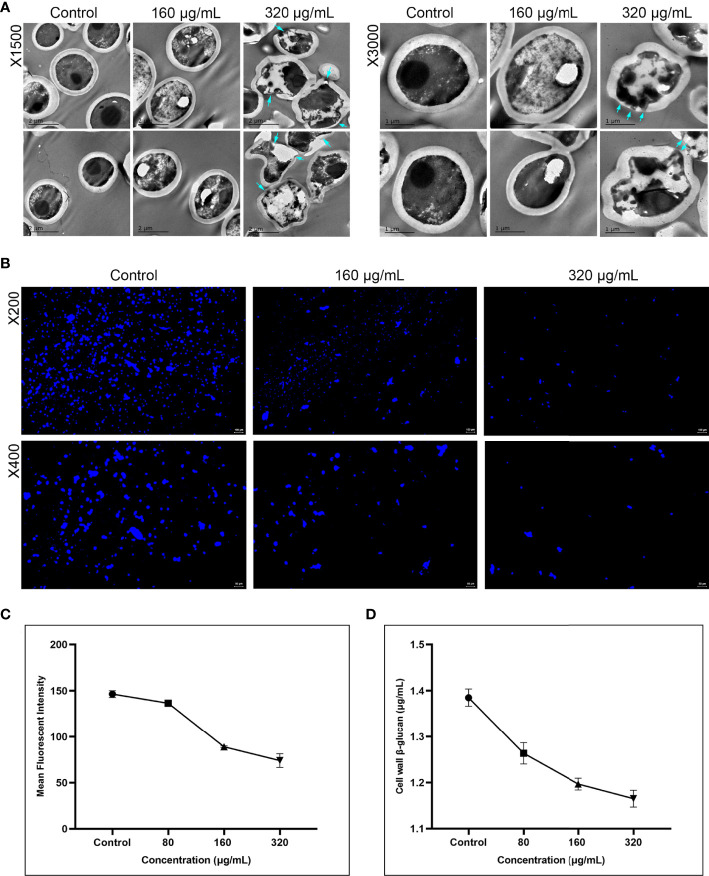
**(A)** The inhibitory effects of magnolol on the cell wall ultrastructure of *C. albicans* assessed using TEM under magnifications of 1500 and 3000. At 320 μg/mL, *C. albicans* ultrastructure showed partial thinning of cell walls and even rupture, with cytoplasmic leakage (blue arrow). **(B)** Magnolol could exert significant antifungal effects by inducing *C. albicans* cell wall stress *via* CFW staining under magnifications of 200 and 400. **(C)** CFW fluorescence quantitative results showed the inhibitory effects of magnolol against *C. albicans* cell walls were concentration-dependent (*P* < 0.001). **(D)** The β-glucan quantitative detection assay showed *C. albicans* cell wall β-glucan content decreased significantly under the action of magnolol in a concentration-dependent manner (*P* < 0.001).

Cell wall integrity and β-glucan synthesis are another two critical virulence factors of *C. albicans* (Lima et al., 2019). Therefore, we further examined the effect of magnolol on the cell wall ultrastructure of *C. albicans* using TEM, CFW staining assay, and β-glucan quantitative detection assay (**Figure 4**). The TEM results showed a significant change in the cell wall morphology and internal structure of *C. albicans* (**Figure 4A**). With an increase in magnolol concentration, *C. albicans* cell walls showed partial thinning, breakage, and even complete disintegration. When magnolol’s concentration reached 320 μg/mL, the cell wall morphology changed significantly, and the cytoplasmic contents underwent crumpling, abnormal distribution, and even leaked out. In addition, the CFW staining and the fluorescence quantification results showed that magnolol could exert antifungal activity by inducing *C. albicans* cell wall stress and defects in a concentration-dependent manner (*P* < 0.001) (**Figure 4B**, **C**). When magnolol’s concentration reached 160 and 320 μg/mL, the fluorescence intensity decreased by 39.22% and 49.45%, respectively, in comparison with the control group. Furthermore, the β-glucan quantitative detection assay showed that *C. albicans* cell wall β-glucan content decreased significantly after drug action, and the inhibitory effects were concentration-dependent (*P* < 0.001) (**Figure 4D**). When the magnolol concentration was 160 and 320 μg/mL, the content of *C. albicans* cell wall β-glucan was only 1.197 and 1.165 μg/mL, respectively. The results above implied that magnolol could inhibit the virulence of *C. albicans* by disrupting its cell wall integrity and impeding the synthesis of cell wall β-glucan.

### Magnolol affected the gene expression of *C. albicans* virulence factors and the related pathways

**Figure 5:**
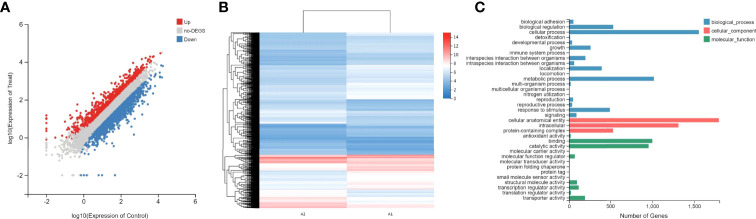
**(A)** Scatter plot of RNA-seq expression analysis. Upregulated genes are marked in red, and downregulated genes are marked in blue. **(B)** Heat map of RNA-seq expression analysis. Genes with statically significant changes were utilized to construct the heat map. The red color indicates relatively high expression, and the blue color indicates relatively low expression. **(C)** GO functional analysis of DEGs based on the RNA-seq data. GO functions are divided into three main categories: biological process (blue), cellular component (red), and molecular function (green). The x-axis represents the number of genes that have the GO function.

**Figure 6:**
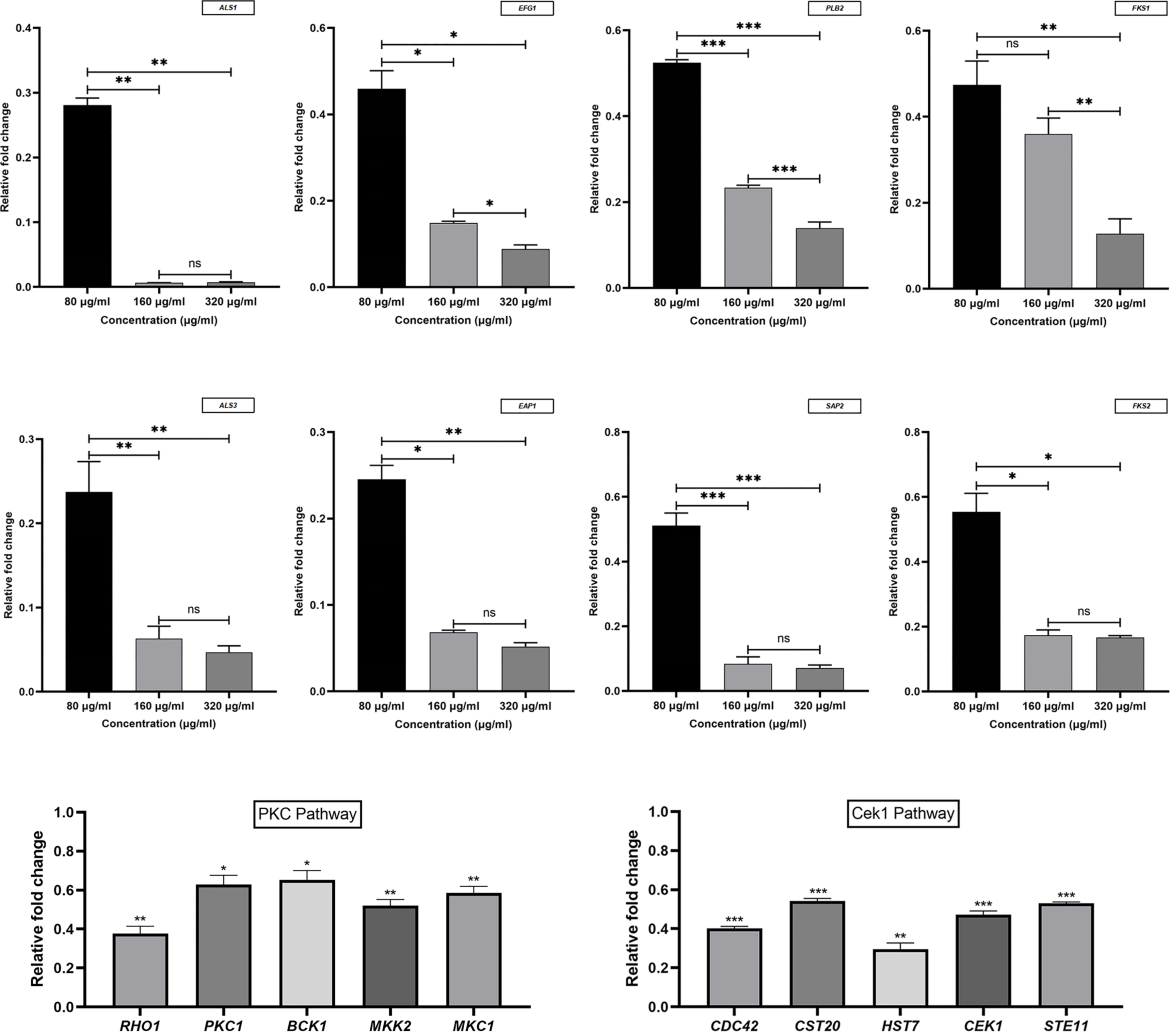
Virulence factors and signaling pathway-related genes of *C. albicans* were measured using qRT-PCR and normalized to the expression of 18S rRNA. Magnolol downregulated the expression of the genes *ALS1*, *ALS3*, *EFG1*, *EAP1*, *PLB2*, *SAP2*, *FKS1*, *FKS2*, *RHO1*, *PKC1*, *BCK1*, *MKK2*, *MKC1*, *CDC42*, *CST20*, *STE11, HST7*, and *CEK1*. *, *P* < 0.05; **, *P* < 0.01; ***, *P* < 0.001; ns, no significance.

To determine the DEGs in response to magnolol against the virulence factors of *C. albicans*, RNA sequencing (RNA-seq) was conducted. At a drug concentration of 160 μg/mL, 1410 genes were upregulated, and 2004 genes were downregulated. We further analyzed these genes for GO annotations, under three categories: biological process, cellular component, and molecular function (**Figure 5**). Based on the three aspects above, we screened out six significantly down-regulated genes related to *C. albicans* virulence factors, including adhesion- and invasion-related genes (*ALS1*, *ALS3*), hyphae- and biofilm-formation-related genes (*EFG1*, *EAP1*), and hydrolase-related genes (*PLB2*, *SAP2*). Furthermore, the down-regulation of cell wall β-1,3-glucan synthesis-related genes (*FKS1*, *FKS2*) and the selected genes above were confirmed using quantitative real-time reverse transcription PCR (qRT-PCR) (**Figure 6**). Under the same drug concentration, the inhibitory effects of magnolol on diverse virulence factor-related genes were different. At 80 μg/mL, magnolol could significantly inhibit the expression of *ALS1*, *ALS3*, and *EAP1*, and as the concentration increased to 160 and 320 μg/mL, magnolol’s inhibitory effects on all the genes above were further enhanced. As for the down-regulated genes of the signaling pathways, PKC pathway-related genes (*RHO1*, *PKC1*, *BCK1*, *MKK2*, *MKC1*), and Cek1 pathway-related genes (*CDC42*, *CST20*, *STE11*, *HST7*, *CEK1*) were also selected *via* RNA-seq and verified by qRT-PCR. The above genes of *C. albicans* virulence factors and the two related pathways were all significantly inhibited by magnolol. Statistical significance was determined by the One-way ANOVA (*P* < 0.05). The results revealed that magnolol could significantly inhibit various *C. albicans* virulence factors, which could be further regulated by PKC and Cek1 signaling pathways.

## Discussion

Magnolol, a lignin compound extracted from *M. officinalis* Cortex, has been reported to have multiple biological activities, such as anti-inflammatory, antioxidant, antibacterial, and antifungal effects (Zhang et al., 2019; Niu et al., 2021). Magnolol has significant inhibitory effects on various bacteria, such as *Streptococcus mutans*, *Porphyromonas gingivali*s, *Fusobacterium nucleatum*, and *Escherichia coli* (Greenberg et al., 2007; Chiu et al., 2021). As for its antifungal activities, early studies showed that magnolol could inhibit *C. albicans* azole efflux and produce synergistic effects with fluconazol (Sun et al., 2015b). In addition, Sun *et al*. found that magnolol could inhibit *C. albicans* in planktonic and biofilm states (Sun et al., 2015a). Moreover, previous research by our group showed that magnolol had a broad-spectrum anti-*Candida* effects, either on the standard or clinically isolated strains of *Candida* spp. (Zhou et al., 2017). However, the antifungal mechanism of magnolol has not been fully investigated. Therefore, we further explored magnolol’s inhibitory effects against *C. albicans* virulence factors and the related pathways in this study.

Biofilm formation, which can be divided into different stages and involves multiple virulence factors, is the critical basis for the pathogenicity of *C. albicans* (Cavalheiro and Teixeira, 2018; Wall et al., 2019). Our previous study have found that magnolol has significant inhibitory effects on several stages of *Candida* biofilm formation (Zhou et al., 2017). Therefore, in this study, we confirmed the role of magnolol against various virulence factors in different stages of biofilm formation. Firstly, in the initial stage of biofilm formation (0–12 h), magnolol could decrease the adhesion of *C. albicans*. Then, in the following stage (12–24 h), the hyphal formation was significantly inhibited by magnolol. After that, with the maturation of the *C. albicans* biofilm (24 h), magnolol-treated biofilms displayed weakened viability and poorly developed architecture. Meanwhile, the *C. albicans* ultrastructure in the biofilms showed partial thinning of cell walls and even rupture with cytoplasm leakage. The cell wall integrity and β-glucan content were also radically reduced.

To compare the effect of diverse concentrations of magnolol against *C. albicans* virulence factors, three concentration gradients, 1/2 BMIC_90_ (80 μg/mL), BMIC_90_ (160 μg/mL), and 2 BMIC_90_ (320 μg/mL), were selected for analysis. Quantitative comparison using qRT-PCR showed that although the concentration of magnolol was relatively low (1/2 BMIC_90_), its inhibitory effects on adhesion, invasion, hyphal formation, and biofilm maturation were fairly prominent. As the concentration increased to the BMIC_90_, its inhibitory effects on the secretion of phospholipase and aspartate protease, as well as the synthesis of cell wall β-1,3 glucan, were greatly enhanced. When the concentration reached 2 BMIC_90_, magnolol’s inhibitory effect on all the above virulence factors became more significant.

**Figure 7:**
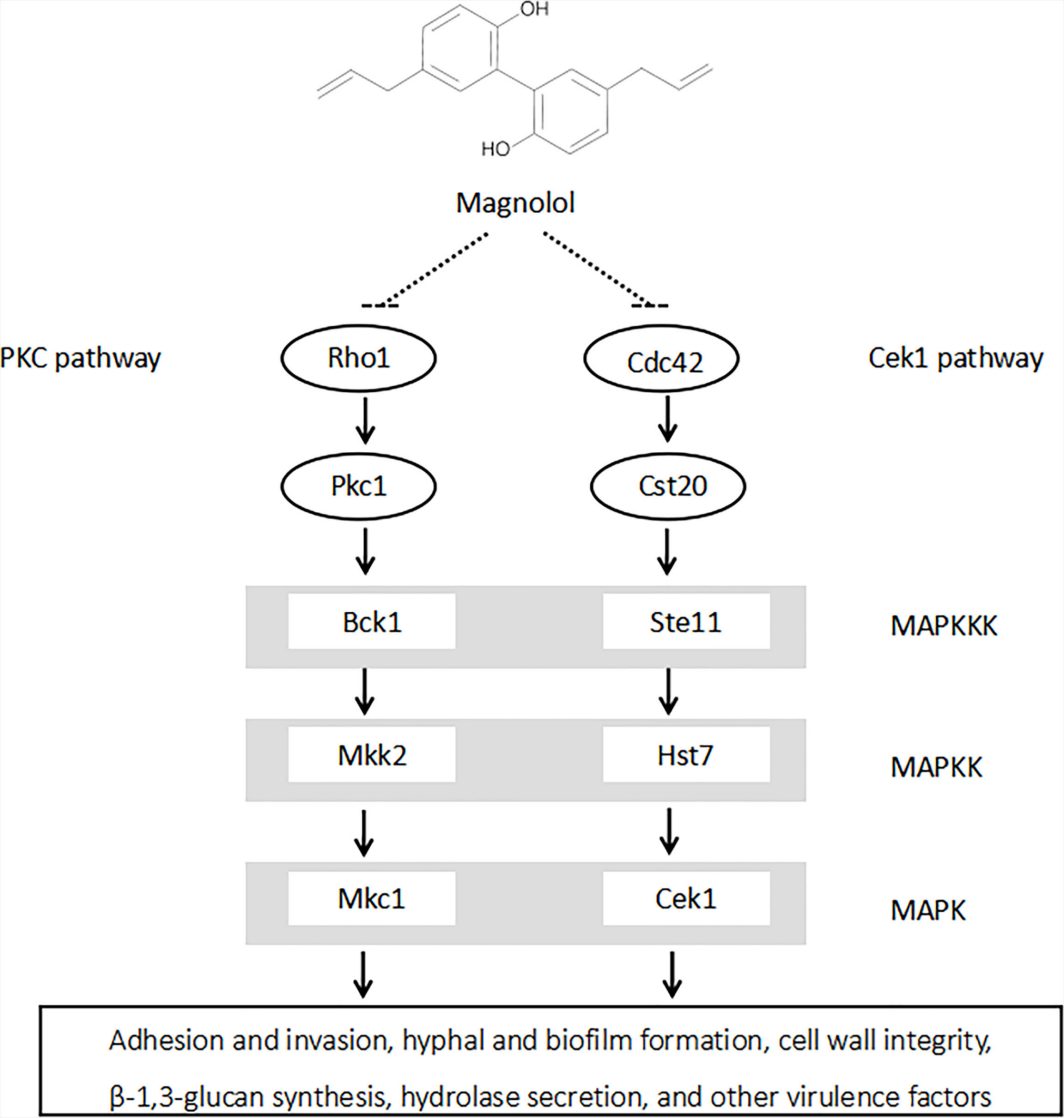
Magnolol could significantly inhibit multiple *C. albicans* virulence factors by down-regulating PKC and Cek1 MAPK signaling pathways.

In terms of signaling pathways, studies have shown that both cAMP and MAPK signaling pathways could play essential roles in the pathogenesis of *C. albicans* (de Dios et al., 2010; Huang et al., 2019). The cAMP signaling pathway is crucial in regulating *C. albicans* morphogenesis and environmental sensing (Huang et al., 2019). The MAPK signaling pathways, including PKC, Cek1, and HOG pathways, are also significant in the regulation of *C. albicans* hyphal formation, biofim formation, and the adaptation to cell wall stress (Schoeters and Van Dijck, 2019). The downregulation of these pathways could further lead to the inhibition of *C. albicans* virulence factors. A previous study showed that the anti-*Candida* effect of magnolol was related to the Ras 1-cAMP-Efg 1 signaling pathway; however, it has not yet been revealed whether magnolol can act through the MAPK signaling pathway (Sun et al., 2015a). In this study, the downregulation of PKC pathway-related genes (*RHO1*, *PKC1*, *BCK1*, *MKK2*, *MKC1*) and Cek1 pathway-related genes (*CDC42*, *CST20*, *STE11*, *HST7*, *CEK1*) were screened out *via* RNA-seq and further confirmed by qRT-PCR. Therefore, it was speculated that magnolol could exert its inhibitory effects against *C. albicans* virulence factors *via* PKC and Cek1 MAPK pathways (**Figure 7**).

The clinical efficacy and safety of different formulae containing magnolol have further been investigated in several clinical trials. Greenberg *et al*. found that subjects using chewing gum containing 2 mg of magnolol and compressed mints containing 4.2 mg of magnolol could effectively relieve oral malodor without any adverse reactions (Greenberg et al., 2007). Campus *et al*. proved that subjects chewing sugar-free gum containing 1.4 or 2.8 mg of magnolol for 5 min 3 times a day (with the total daily intake of magnolol being 7 mg/day) showed beneficial effects on oral health, including a reduction in salivary *mutans streptococci*, plaque acidogenicity, and bleeding upon probing, and no adverse reactions were observed in the subjects (Campus et al., 2011). Wessel *et al*. also found that no volunteers showed any adverse effects after chewing sorbitol-containing gum with 6 mg of Magnolia bark extract (containing approximately 94% magnolol) added 3 times daily for up to 4 weeks of medication (Wessel et al., 2017). In the present study, the magnolol concentrations selected were 80 – 320 μg/mL, and the amount of magnolol in each experimental unit was only 0.16 – 0.64 mg, which was far less than the amounts used in the clinical trials mentioned above. Therefore, the concentration of magnolol in our present study is relatively safe for topical administration, allowing for its clinical employment as topical agents, including magnolol-added lozenges, mouthwash, toothpaste, etc., in the prevention and treatment of oral candidiasis in the future. However, more high-quality clinical trials are still needed to confirm the efficacy and safety of magnolol in the future.

## Conclusion

In the present study, we comprehensively explored the inhibition mechanism of magnolol against *C. albicans* virulence factors and the related signaling pathways. The originality of our study lies in three aspects. First, we confirmed the role of magnolol against a variety of virulence factors in different stages of biofilm formation. Second, we determined that the anti-*Candida* effects of magnolol were associated with inhibiting the cell wall integrity, β-1,3-glucan synthesis, and hydrolase secretion, which has not been identified as magnolol’s targeted virulence factors in previous studies. Third, we identified the PKC and Cek1 MAPK signaling pathways for the first time as two novel possible pathways for magnolol in regulating multiple *C. albicans* virulence factors. The findings of this study lay the theoretical foundation for future research into the activities of magnolol against *C. albicans in vivo*, and suggest its promising clinical applications in *Candida* infections.

## Data availability statement

The RNA-seq raw sequencing data have been deposited in the NCBI’s Sequence Read Archive (SRA) under the accession number PRJNA791518. [https://www.ncbi.nlm.nih.gov/bioproject/PRJNA791518/].

## Author contributions

YX performed the methodology, software, investigation, resources, data curation, and writing original draft preparation. HH contributed to the conceptualization, methodology, validation, formal analysis, resources, data curation, supervision, project administration, review and editing. PZ were responsible for the conceptualization, methodology, validation, formal analysis, investigation, resources, data curation, supervision, project administration, funding acquisition, review and editing. All authors have read and agreed to the published version of the manuscript.

## Funding

This research was funded by the Beijing Natural Science Foundation (grant number 7204330) and the Program for New Clinical Techniques and Therapies of Peking University School and Hospital of Stomatology (grant number PKUSSNCT-21B13).

## Conflict of interest

The authors declare that the research was conducted in the absence of any commercial or financial relationships that could be construed as a potential conflict of interest.

## Publisher’s note

All claims expressed in this article are solely those of the authors and do not necessarily represent those of their affiliated organizations, or those of the publisher, the editors and the reviewers. Any product that may be evaluated in this article, or claim that may be made by its manufacturer, is not guaranteed or endorsed by the publisher.
