# Supplementary material for: Magnolol as a potent antifungal agent inhibits Candida albicans virulence factors via the PKC and Cek1 MAPK signaling pathways
Source: Front Cell Infect Microbiol. 2022 Jul 22;12:935322. doi: 10.3389/fcimb.2022.935322 (PMC9355038; doi:10.3389/fcimb.2022.935322)
Supplement: Supplementary file 1 [file Table_1.doc]

**Table S1.** Primer sequences for qRT-PCR analyses.

| **Gene name** |  | **Primer sequences (5′ to 3′)** |
| --- | --- | --- |
| 18S rRNA | Forward | 5’- AATTACCCAATCCCGACAC-3’ |
|  | Reverse | 5’- TGCAACAACTTTAATATACGC-3’ |
| *ALS1* | Forward | 5’- TTGGGTTGGTCCTTAGATGG-3’ |
|  | Reverse | 5’- ATGATTCAAAGCGTCGTTC-3’ |
| *ALS3* | Forward | 5’- CTAATGCTGCTACGTATAATT-3’ |
|  | Reverse | 5’- CCTGAAATTGACATGTAGCA-3’ |
| *EFG1* | Forward | 5’- TATGCCCCAGCAAACAACTG-3’ |
|  | Reverse | 5’- TTGTTGTCCTGCTGTCTGTC-3’ |
| *EAP1* | Forward | 5’- CTGCTCACTCAACTTCAATTGTCG-3’ |
|  | Reverse | 5’- GAACACATCCACCTTCGGGA-3’ |
| *PLB2* | Forward | 5’- GCTTCCATTGATCCACCGTAA-3’ |
|  | Reverse | 5’- GCAATGCCATCTCCTTGTGA-3’ |
| *SAP2* | Forward | 5’- CAAGTGGTTCATCAGCTTCAC-3’ |
|  | Reverse | 5’- TTATTTGTCCCGTGGCAG-3’ |
| *FKS1* | Forward | 5’- GCCGTACATTTGATGTCATT-3’ |
|  | Reverse | 5’- AAAGTACCAGAAGTGACAAC-3’ |
| *FKS2* | Forward | 5’- GATCACGAGTCTGTGATTG-3’ |
|  | Reverse | 5’- AATACATGAGACCAGCCTC-3’ |
| *RHO1* | Forward | 5’- TCAAGGTGTTCCAATCATTTTAGT-3’ |
|  | Reverse | 5’- ACATTCCAAGTAATCAGCAGCA-3’ |
| *PKC1* | Forward | 5’- TGTGCCCTTTGTGGAGAGTT-3’ |
|  | Reverse | 5’- CAGCATCATAATCGGAGCCT-3’ |
| *BCK1* | Forward | 5’- GAAAAGCAACGGTATTCTAAGCA-3’ |
|  | Reverse | 5’- CAGCATCATAATCGGAGCCT-3’ |
| *MKK2* | Forward | 5’- ACGAAAACCACCACCAATAGAT-3’ |
|  | Reverse | 5’- GTTCCAAATGATGCTTTACCG-3’ |
| *MKC1* | Forward | 5’- TGGCGTTGGATTTATTGGA-3’ |
|  | Reverse | 5’- TCCAACCCATCTACAGTCTCAA-3’ |
| *CDC42* | Forward | 5’- AATTACCCAATCCCGACAC-3’ |
|  | Reverse | 5’- ACAGGAGGTTCTAATGCAG-3’ |
| *CST20* | Forward | 5’- ACTGGGGAAACTAATGATAGGACT-3’ |
|  | Reverse | 5’- CATTGTCACTATCATCCCCTTG-3’ |
| *HST7* | Forward | 5’- TCATCAGCTTCTTCTATAC-3’ |
|  | Reverse | 5’- TATTGAGGAAATGACAGTT-3’ |
| *CEK1* | Forward | 5’- CAGGCTCAGGCTCAAGCTCA-3’ |
|  | Reverse | 5’- GGCTGCTGCCTGTTGTTGTT-3’ |
| *STE11* | Forward | 5’- GTCCATTCAGTTCCACACGG-3’ |
|  | Reverse | 5’- TCAATGGCTGCTGGCGTA-3’ |

*Abbreviations: qRT-PCR, quantitative real-time reverse transcription PCR; ALS, Agglutinin-like protein; EFG, Enhanced filamentous growth protein; EAP, Enhanced aherence to polystyrene; PLB, Phospholipases B; SAP, Secretory aspartyl proteinases; PKC, Protein kinase C.
